# Supplementary material for: White matter disconnection of left multiple demand network is associated with post-lesion deficits in cognitive control
Source: Nat Commun. 2023 Mar 29;14:1740. doi: 10.1038/s41467-023-37330-1 (PMC10060223; doi:10.1038/s41467-023-37330-1)
Supplement: Supplementary file 1 — Supplementary Information [file 41467_2023_37330_MOESM1_ESM.pdf]

Supplementary Materials

White matter disconnection of left multiple demand network is associated with post-lesion deficits in cognitive control

Jiefeng Jiang[1,2,3], Joel Bruss[4,5], Woo-Tek Lee[1,2,6], Daniel Tranel[1,3,4] and Aaron D. Boes[3,4,5,7]

[1]Department of Psychological and Brain Sciences, University of Iowa, Iowa City, IA 52242, USA
[2]Cognitive Control Collaborative, University of Iowa, Iowa City, IA 52242, USA
[3]Iowa Neuroscience Institute, University of Iowa, Iowa City, IA 52242, USA
[4]Department of Neurology (Division of Neuropsychology and Cognitive Neuroscience), Carver College of Medicine, Iowa City, IA, 52242, USA
[5]Department of Psychiatry, Carver College of Medicine, Iowa City, IA 52242, USA
[6]Behavioral-biomedical Interface Training Program, University of Iowa, Iowa City, IA 52242, USA
[7]Department of Pediatrics, Carver College of Medicine, Iowa City, IA 52242, USA

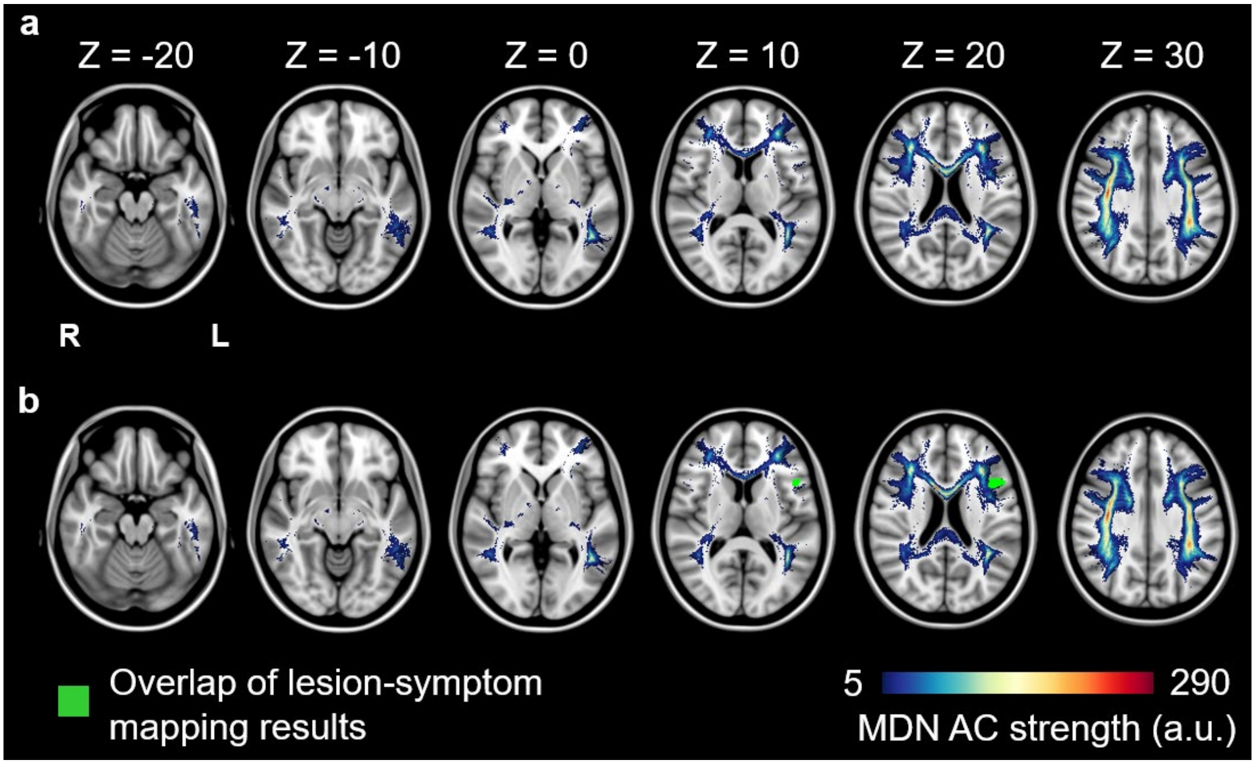

Supplementary Figure 1. Diffusion tensor imaging (DTI) tractography of the Multiple demand network (MDN) and its overlap with lesion-symptom mapping results. (a) MDN anatomical connectivity (AC) connectivity map. For each voxel, its color encodes the value of the first principal component from a principal component analysis performed on brain-wide deterministic tractography results seeded from each cortical region of interest (ROI) within the MDN. Higher values indicate stronger anatomical connectivity between the voxel and the cortical regions of the MDN. (b) MDN AC connectivity map overlaid with the overlap of trail-making task (TMT) and Stroop lesion-symptom mapping results (in green).

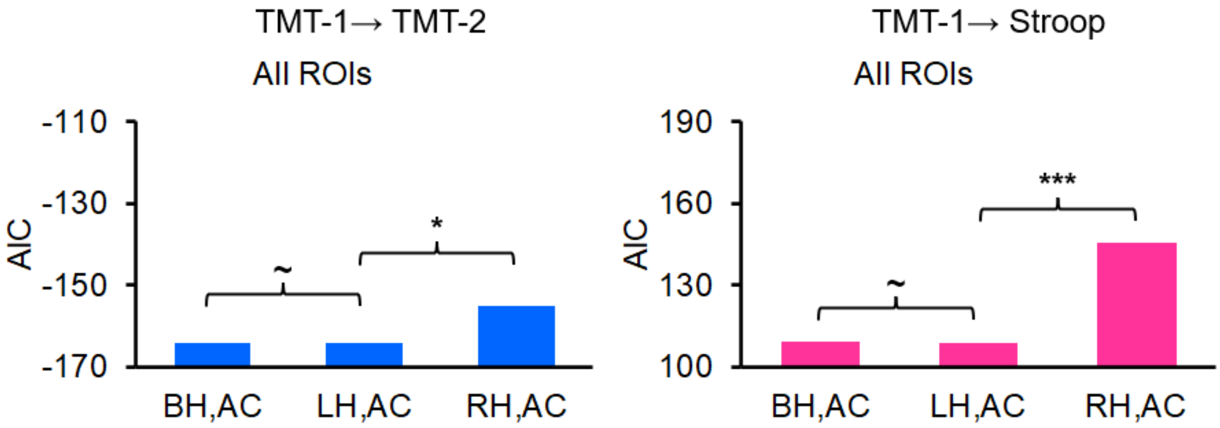

Supplementary Figure 2. Prediction performance varies based on lesion laterality. We performed a secondary analysis to evaluate if balancing the sample size between right versus left-sided lesions would influence the finding that left hemisphere (LH) lesions explain more variance in cognitive control performance. P-values are based on one-sided tests of Akaike information criterion (AIC) difference (see Methods). Balancing was based on a paired t-test comparing LH vs. right hemisphere (RH) lesion volume across subjects in each sample. Trail-making task-1 (TMT-1) was already balanced ($p > 0.93$) thus no additional analysis was conducted. For samples TMT-2 and Stroop, we removed subjects with highest LH-RH lesion volumes until the p-value of the paired t-test maximized (TMT-2: $p > 0.99$; Stroop: $p > 0.95$). The resulting samples had 268 and 214 participants for TMT-2 and Stroop, respectively. Consistent with the findings from the main analysis, behavioral scores were better predicted by LH lesion scores than RH (TMT-1 → TMT-2: $\Delta AIC = -8.9$, $p = 0.012$, $R = 1.02$; TMT-1 → Stroop: $\Delta AIC = -36.9$, $p = 1\times10^{-8}$, $R = 1.09$). Similar to the main analysis, prediction performance did not significantly improve by adding RH lesion scores to LH lesion scores (both ps > 0.39). *: $p < 0.05$; ***: $p < 0.001$; ~: $p > 0.05$.

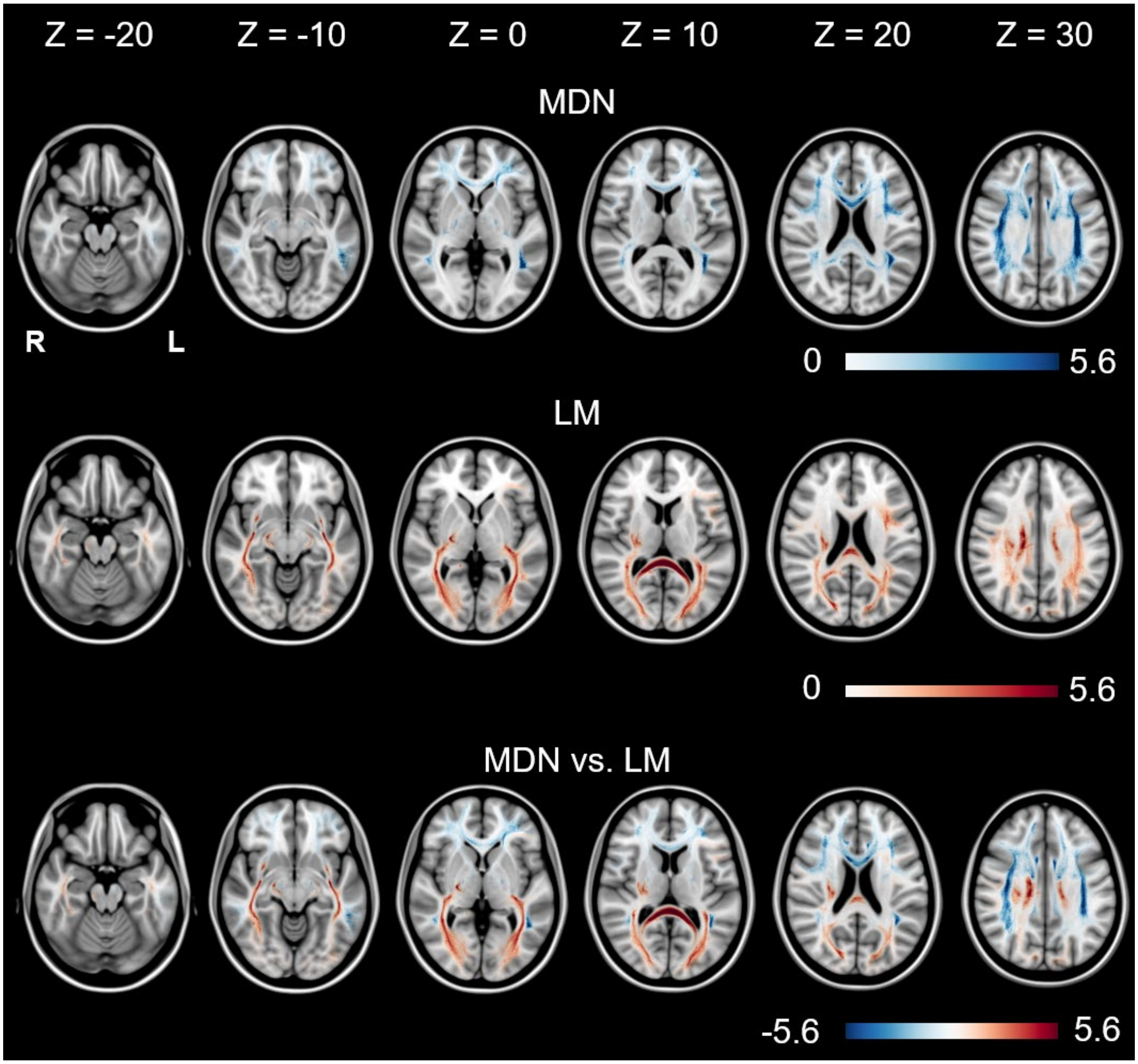

Supplementary Figure 3. Anatomical connectivity (AC) patterns of the multiple demand network (MDN, top) and lesion-symptom mapping (LM, middle) and the difference in AC patterns between MDN and LM (bottom). For each network (MDN or LM), the AC maps seeding from each of the regions of interest (ROIs) are averaged and color-coded. Color encodes average streamline numbers from tractography (see Methods). Compared to the LM, MDN AC maps show more anterior connectivity and less connectivity linking inferior parietal and temporal areas.

Etiology	TMT-1	TMT-2	Stroop
Stroke / Ischemic	182	154	102
Stroke / Hemorrhagic	66	41	31
Subarachnoid hemorrhage with intraparenchymal lesion	12	14	16
Tumor resection	40	43	45
Resection of arteriovenous malformation, cavernoma, cyst	10	13	13
Resection, Abscess	1	1	1
Trauma with focal intraparenchymal lesion	13	10	11
Encephalitis, herpes simplex	8	5	5
Encephalitis, limbic or other	2	1	1
Other	1	5	4
Total	335	287	229

Supplementary Table 1. Summary of Etiology in each of the three samples.

Supplementary Note 1. Testing construct validity of TMT and the Stroop task

We define cognitive control as the top-down modulation on neural processing to achieve goal-directed behaviors, consistent with its use in the literature[1,2]. Previous neuropsychological studies have shown that the TMT B-A scores[3,4] and the Stroop interference scores[5] capture cognitive control relatively cleanly, as they are contrast scores that attempt to control for inter-individual behavioral differences that rely on processes other than cognitive control that could also influence performance (e.g. motor control, understanding instructions, visual processing, processing speed). To assess the construct validity of these measures in this study, we took a discriminant validity approach. That is, we tested whether variability in TMT B-A and Stroop interference scores can be explained by classic measures of other cognitive processes. Specifically, we chose three measures, namely the vocabulary score from the 4th edition of the Wechsler Adult Intelligence Scale (WAIS-IV), the symbol search score from the WAIS-IV, and the Judgment of Line Orientation score[6], as measures of verbal naming, processing and visuospatial abilities, respectively. The measures were chosen based on their availability in the database and their relevance to the TMT and Stroop tasks (i.e., the Stroop task involves words of color; the TMT requires subjects to search for letters and/or digits, similar to the symbol search task of the WAIS-IV; and both tasks require basic visual processing).

Note that the three selected measures may also be confounded by cognitive control when they are used to analyze individual differences. For example, higher cognitive control can enhance the processing of task-relevant information and improve the suppression of distractors (e.g., distractors from the task, other internal thoughts and/or the testing environment) and can thus benefit subjects in all tests. Thus, to test whether our measures are confounded by each of the three selected measures while controlling for potential shared variance related to cognitive control, for each selected measure (e.g., vocabulary score of the WAIS-IV) and each of the two cognitive control measures (e.g., TMT B-A), we performed a linear regression analysis using the former to predict the latter, while using the other cognitive control measure (e.g., Stroop interference score) as a nuisance predictor to control for shared variance due to cognitive control. None of the three selected measures displayed statistically significant effect in explaining variances in the two measures of cognitive control ($n = 72$-204, depending on the availability of test scores; all $ps > 0.08$ after FDR correction). The findings from the discriminant validity analysis suggest that the TMT B-A and the Stroop interference score cannot be explained by impaired performance in these other related processes and are consistent with the results capturing performance related to cognitive control.

Supplementary Note 2. Behavioral analysis

In TMT-1, part A scores (mean ± SD: 42.8 ± 24.0s) were significantly lower than part B scores (111.4 ± 75.5s; $t_{334} = -20.82$, $p = 3\times10^{-62}$, Cohen's d = 1.14). Similarly, in TMT-2, part A scores (36.8 ± 19.2s) were significantly lower than part B scores (90.4 ± 49.6s; $t_{286} = -23.87$, $p = 5\times10^{-70}$, Cohen's d = 1.41). In Stroop sample, the score in color-word test (33.8 ± 11.6) is lower than that in color (64.7 ± 16.0) and word tests (87.3 ± 20.7), as reflected in an interference score significantly below zero (defined as CW–(C+W)/2; -42.2 ± 12.3, $t_{228} = -51.89$, $p = 3\times10^{-128}$, Cohen's d = 3.43). In sum, in all samples, we observed strong evidence showing slower and\or worse performance in tests requiring more cognitive control (e.g., TMT part B and the color-word Stroop condition), thus supporting the notion that our behavioral measures capture cognitive control.

Supplementary Note 3. Testing the correlation between cognitive control performance and co-occurring symptoms

To address the possibility of co-occurring symptoms confounding prediction performance, we tested correlations (Spearman's ρ was used to account for non-normal distributions of some test scores) between neuropsychological scores reflecting five potential co-occurring symptoms and behavioral scores of cognitive control (TMT and Stroop). First, the aphasia severity rating scores from the Boston Diagnostic Aphasia Examination did not correlate with TMT ($n = 135$, $\rho = 0.014$, $p > 0.91$) or Stroop scores ($n = 64$, $\rho = 0.029$, $p > 0.81$). Second, visual neglect ratings did not correlate with TMT ($n = 171$, $\rho = -0.081$, $p > 0.54$) or Stroop scores ($n = 58$, $\rho = -0.10$, $p > 0.45$). Third, to test the confound of hemiplegia, we used grooved pegboard test scores on left and right hand separately. The left minus right contrast was used as a measure of asymmetry in psychomotor performance and was not correlated with TMT ($n = 294$, $\rho = -0.047$, $p > 0.66$) or Stroop scores ($n = 89$, $\rho = 0.073$, $p > 0.49$). The absolute value of this contrast was also employed as a measure of hemiplegia on either side of the body. This measure was not correlated with TMT ($n = 294$, $\rho = 0.007$, $p > 0.94$) or Stroop scores ($n = 89$, $\rho = -0.14$, $p > 0.18$). Lastly, the average of left- and right-hand grooved pegboard test scores was used as a measure of slowing in processing and potential fatigue and did not correlate with TMT ($n = 294$, $\rho = -0.051$, $p > 0.63$) or Stroop scores ($n = 89$, $\rho = -0.063$, $p > 0.55$). For completeness, we also correlated left- and right-hand grooved pegboard test scores separately with TMT (left: $n = 307$, $\rho = -0.094$, $p > 0.36$; right: $n = 299$, $\rho = -0.064$, $p > 0.54$) and Stroop (left: $n = 93$, $\rho = -0.068$, $p > 0.51$; right: $n = 91$, $\rho = -0.098$, $p > 0.35$) scores and did not observe significant correlations. Taken together, we did not find evidence that the cognitive control performance scores were explained by these common co-occurring symptoms.

Supplementary Note 4. Testing the WM connectivity of the MDN's overlap with lesion-symptom maps

We tested whether lesion locations that are associated with impaired cognitive control (based on their inclusion in the significant multivariate lesion-symptom maps) are located in the WM tracks linking the MDN. This approach provides a complementary approach for evaluating the anatomy most critical for cognitive control in relation to the MDN anatomy. Specifically, we tested whether a lesion-symptom map overlaps the MDN AC map at above chance level by comparing the size of the overlap to a null distribution of random overlaps. The null distribution was constructed by randomly permuting the locations of the clusters in the lesion-symptom map within a WM mask for 1,000 times. After each permutation, the size of the overlap of the permuted clusters and the MDN AC map was computed as the summation of the voxel-wise dot product of connectivity strength in the MDN AC map and the clusters' lesion load in the lesion-symptom map. The overlap sizes were pooled as an estimate of the null distribution to derive the p value of the statistical test.

Using the approach above, we found that the conjunction of the TMT and the Stroop lesion-symptom maps (Figure 2B) significantly overlaps with the WM tracks linking the MDN ($p = 0.03$). The overlap is driven by the TMT lesion-symptom map ($p = 0.03$) compared to the Stroop lesion-symptom-map ($p = 0.26$), possibly due to the larger sample size in the former. The lesion-symptom map using the latent variable (Figure 2D) did not significantly overlap with the WM tracks linking the MDN ($p = 0.19$). Despite the Stroop and latent variable maps not reaching significance both intersected with the MDN WM map in the frontal lobe.

Supplementary Note 5. Sampling and testing procedure

The sampling procedure involves screening individuals with focal, acquired intraparenchymal lesions that are otherwise neurological and psychiatrically healthy. Many individuals are identified when they are referred for neuropsychological testing. As such, it is possible that this cohort is more enriched in individuals with some degree of cognitive impairment that prompted the referral. In addition, we only enroll patients that are able to participate in cognitive testing, which eliminates individuals with certain types of lesions that result in coma or more severe disability. There is no bias in sampling with regard to the specific hypotheses tested here. Note that we included subjects with different types of lesions (Supplementary Table 1) to increase the sample size and generalizability of the findings. Different etiologies of acquired brain lesions are each associated with specific limitations, such as ischemic stroke lesions being limited to vascular distributions. Inclusion of multiple different lesion etiologies is one way to overcome the limitations of over-reliance on any one etiology. However, one limitation of this approach is that different types of lesions may have different mechanisms of recovery and/or plasticity, which may in turn impede the prediction performance of the model. We acknowledge there is no perfect solution, but we are of the opinion that higher sample sizes with diverse etiologies is preferred relative to smaller samples with a single etiology. When more data is available, future research is encouraged to investigate the similarity of and difference between cognitive control recovery following different types of lesions.

The TMT is given to all subjects as part of the "core" neuropsychological test battery upon enrollment in the Registry. The Stroop task is given less commonly and is often utilized as a follow-up assessment if impairments in executive function are observed within the core battery. There is no systemic bias in patient selection in terms of lesion location.

Supplementary References

1 Egner, T. *The Wiley handbook of cognitive control*. (Wiley Blackwell, 2017).

2 Miller, E. K. & Cohen, J. D. An integrative theory of prefrontal cortex function. *Annual review of neuroscience* **24**, 167-202 (2001).

3 Bowie, C. R. & Harvey, P. D. Administration and interpretation of the Trail Making Test. *Nature protocols* **1**, 2277-2281 (2006).

4 Sánchez-Cubillo, I. *et al.* Construct validity of the Trail Making Test: role of task-switching, working memory, inhibition/interference control, and visuomotor abilities. *Journal of the International Neuropsychological Society* **15**, 438-450 (2009).

5 Scarpina, F. & Tagini, S. The stroop color and word test. *Frontiers in psychology* **8**, 557 (2017).

6 Mitrushina, M., Boone, K. B., Razani, J. & D'Elia, L. F. *Handbook of normative data for neuropsychological assessment*. (Oxford University Press, 2005).
