# Supplementary material for: White matter disconnection of left multiple demand network is associated with post-lesion deficits in cognitive control
Source: Nat Commun. 2023 Mar 29;14:1740. doi: 10.1038/s41467-023-37330-1 (PMC10060223; doi:10.1038/s41467-023-37330-1)
Supplement: Supplementary file 3 — Reporting Summary [file 41467_2023_37330_MOESM3_ESM.pdf]

nature portfolio

Corresponding author(s): Jiefeng Jiang
Aaron D. Boes

Last updated by author(s): Feb 12, 2022

Reporting Summary

Nature Portfolio wishes to improve the reproducibility of the work that we publish. This form provides structure for consistency and transparency in reporting. For further information on Nature Portfolio policies, see our Editorial Policies and the Editorial Policy Checklist.

Statistics

For all statistical analyses, confirm that the following items are present in the figure legend, table legend, main text, or Methods section.

n/a	Confirmed	
☐	☒	The exact sample size (n) for each experimental group/condition, given as a discrete number and unit of measurement
☐	☒	A statement on whether measurements were taken from distinct samples or whether the same sample was measured repeatedly
☐	☒	The statistical test(s) used AND whether they are one- or two-sided<br>*Only common tests should be described solely by name; describe more complex techniques in the Methods section.*
☐	☒	A description of all covariates tested
☐	☒	A description of any assumptions or corrections, such as tests of normality and adjustment for multiple comparisons
☐	☒	A full description of the statistical parameters including central tendency (e.g. means) or other basic estimates (e.g. regression coefficient) AND variation (e.g. standard deviation) or associated estimates of uncertainty (e.g. confidence intervals)
☐	☒	For null hypothesis testing, the test statistic (e.g. F, t, r) with confidence intervals, effect sizes, degrees of freedom and P value noted<br>*Give P values as exact values whenever suitable.*
☒	☐	For Bayesian analysis, information on the choice of priors and Markov chain Monte Carlo settings
☒	☐	For hierarchical and complex designs, identification of the appropriate level for tests and full reporting of outcomes
☐	☒	Estimates of effect sizes (e.g. Cohen's d, Pearson's r), indicating how they were calculated

Our web collection on statistics for biologists contains articles on many of the points above.

Software and code

Policy information about availability of computer code

Data collection	No software was used for data collection
Data analysis	R package LESYMAP, DTI studio, LEAD-DBS pipeline, FSL and Matlab 2020a. Analysis code is shared at https://github.com/JiefengJiang/CPMCognitiveControl

For manuscripts utilizing custom algorithms or software that are central to the research but not yet described in published literature, software must be made available to editors and reviewers. We strongly encourage code deposition in a community repository (e.g. GitHub). See the Nature Portfolio guidelines for submitting code & software for further information.

Data

Policy information about availability of data

All manuscripts must include a data availability statement. This statement should provide the following information, where applicable:

- Accession codes, unique identifiers, or web links for publicly available datasets
- A description of any restrictions on data availability
- For clinical datasets or third party data, please ensure that the statement adheres to our policy

Lesion masks and neuropsychological test data are available under restricted access, subject to the policies and procedures of the Iowa Cognitive Neuroscience Patient Registry and the Benton Neuropsychology Clinic. Data access may be requested by emailing A.D.B and will be replied within a month. Source data are provided with this paper.

Public datasets: Human Connectome project, rs-fcMRI

Human research participants

Policy information about studies involving human research participants and Sex and Gender in Research.

Reporting on sex and gender	See Table 1
Population characteristics	See Table 1 and Supplementary Table 1
Recruitment	Participants were recruited by the Department of Neurology at the University of Iowa. The sampling procedure involves screening individuals with focal, acquired intraparenchymal lesions that are otherwise neurological and psychiatrically healthy. Many individuals are identified when they are referred for neuropsychological testing. As such, it is possible that this cohort is more enriched in individuals with some degree of cognitive impairment that prompted the referral. In addition, we only enroll patients that are able to participate in cognitive testing, which eliminates individuals with certain types of lesions that result in coma or more severe disability. There is no bias in sampling with regard to the specific hypotheses tested here. Note that we included subjects with different types of lesions (Supplementary Table 1) to increase the sample size and generalizability of the findings. Different etiologies of acquired brain lesions are each associated with specific limitations, such as ischemic stroke lesions being limited to vascular distributions. Inclusion of multiple different lesion etiologies is one way to overcome the limitations of over-reliance on any one etiology. However, one limitation of this approach is that different types of lesions may have different mechanisms of recovery and/or plasticity, which may in turn impede the prediction performance of the model. We acknowledge there is no perfect solution, but we are of the opinion that higher sample sizes with diverse etiologies is preferred relative to smaller samples with a single etiology. When more data is available, future research is encouraged to investigate the similarity of and difference between cognitive control recovery following different types of lesions.<br>The TMT is given to all subjects as part of the "core" neuropsychological test battery upon enrollment in the Registry. The Stroop task is given less commonly and is often utilized as a follow-up assessment if impairments in executive function are observed within the core battery. There is no systemic bias in patient selection in terms of lesion location.
Ethics oversight	Prior to any data collection, this study was approved by the Institutional Review Board of the University of Iowa. Participants recruited through the Patient Registry signed informed consent prior to performing cognitive testing while de-identified clinical data from participants from the Benton Neuropsychology Clinic were acquired by chart review of retrospective electronic medical records following approval by the Institutional Review Board.

Note that full information on the approval of the study protocol must also be provided in the manuscript.

Field-specific reporting

Please select the one below that is the best fit for your research. If you are not sure, read the appropriate sections before making your selection.

☐ Life sciences ☒ Behavioural & social sciences ☐ Ecological, evolutionary & environmental sciences

For a reference copy of the document with all sections, see nature.com/documents/nr-reporting-summary-flat.pdf

Behavioural & social sciences study design

All studies must disclose on these points even when the disclosure is negative.

Study description	Quantitative correlational study.
Research sample	Patients with focal lesions from the Iowa Neurology Registry and the Benton Neuropsychology Clinic. Demographical information is reported in Table 1. The sample is representative in the area of Iowa City, IA.
Sampling strategy	Convenience sample from the Iowa Neurology Registry and the Benton Neuropsychology Clinic. All participants with both neuroimaging lesion masks and neuropsychological test scores for the Trail Making task and the Stroop task were included in the study.
Data collection	We utilized two different tasks to assess cognitive control performance. TMT and Stroop task. Both tasks were administrated by trained researchers. Participants completed both tasks on paper in a room with a trained researcher. In rare cases, a second researcher was present for training purposes.<br><br>TMT consisted of part A and B. In part A (TMT A), participants were required to link 25 circles, each of which contains a number from 1 to 25, by drawing lines in ascending order of number values as fast as possible. In part B (TMT B), participants encountered circles containing either a number or a letter, and were required to link the circles by drawing lines in ascending order, and at the same time, alternating between number and letter circles (1-A-2-B-3-C...), as fast as possible.

The Stroop test consisted of three different conditions: color (C), word (W), and color-word (CW). In the C condition, patients were presented squares painted in either red, blue or green color and were asked to name the color of squares. In the W condition, patients were asked to read the words that are written in black ink, and words could be either 'red', 'blue', or 'green'. Lastly, in the CW condition, patients saw the words 'red', ''blue', 'green', but printed in incongruent color (e.g., word 'red' in green ink color), and are required to state the color of the ink for the word.

Each participant included in the analysis had a focal brain lesion with visible boundaries evident from structural imaging sequences on MRI. CT scans were used in rare cases when MRI was contraindicated (n=64). Lesions were manually segmented in three dimensions by a rater blind to behavioral test scores, and anatomical accuracy of each tracing was reviewed by a neurologist (A.D.B.) in both native space and upon transformation to MNI152 1-mm template brain using a combination of linear and nonlinear registration techniques. The neurologist was blinded to the experimental groups.

Timing: The first subject used in this study was scanned in July, 1992. The latest participant was scanned in August, 2021.

Data exclusions: No data was excluded.

Non-participation: As this study uses existing data, non-participation information is unknown.

Randomization: Subjects were divided into 3 groups such that no subject was in both the training and test data of the same analysis.

Reporting for specific materials, systems and methods

We require information from authors about some types of materials, experimental systems and methods used in many studies. Here, indicate whether each material, system or method listed is relevant to your study. If you are not sure if a list item applies to your research, read the appropriate section before selecting a response.

Materials & experimental systems

n/a	Involved in the study	
☒	☐	Antibodies
☒	☐	Eukaryotic cell lines
☒	☐	Palaeontology and archaeology
☒	☐	Animals and other organisms
☒	☐	Clinical data
☒	☐	Dual use research of concern

Methods

n/a	Involved in the study	
☒	☐	ChIP-seq
☒	☐	Flow cytometry
☐	☒	MRI-based neuroimaging

Magnetic resonance imaging

Experimental design

Design type: Structural MRI

Design specifications: N/A

Behavioral performance measures: N/A

Acquisition

Imaging type(s): Structural MRI

Field strength: As the data acquisition spans over 30 years, the field strength and imaging protocols changes over time. Nevertheless, we do not expect that this variability would change the key findings, as all lesion masks were manually traced by trained neurologists.

Sequence & imaging parameters: See above

Area of acquisition: whole brain

Diffusion MRI ☐ Used ☒ Not used

Preprocessing

Preprocessing software: FSL

Normalization: non-linear transformation

Normalization template: MNI152

Noise and artifact removal: Lesions were manually traced by a neurologist.

Volume censoring: N/A

Statistical modeling & inference

Model type and settings: Correlation between structural neuroimage and behavioral performance.

Effect(s) tested: Model performance in Akaike information criterion (AIC)

Specify type of analysis: ☐ Whole brain ☒ ROI-based ☐ Both

Anatomical location(s): ROIs were defined by the HCP atlas.

Statistic type for inference (See Eklund et al. 2016): Inference was made based on cross-validation or cross-sample tesing.

Correction: FDR

Models & analysis

n/a	Involved in the study
☐	☒ Functional and/or effective connectivity
☒	☐ Graph analysis
☐	☒ Multivariate modeling or predictive analysis

Functional and/or effective connectivity: For each ROI, its FC network was constructed using rs-fcMRI. The primary rs-fcMRI dataset included 98 healthy right-handed subjects (48 male subjects, age 22 ± 3.2 years), that were resting quietly at the time of data collection. These data are part of a larger, publicly available data set used previously. Rs-fcMRI data were processed in accordance with previously described methods. Participants completed two 6.2 min rs-fcMRI scans during which they were asked to rest in the scanner (3T, Siemens) with their eyes open (TR = 3,000 ms, TE = 30 ms, FA = 85, 3 mm voxel size [27 mm3], FOV = 216, 47 axial slices with interleaved acquisition and no gap). Functional data were spatially smoothed using a Gaussian kernel of 4 mm full-width at half-maximum. The data were temporally filtered (.009 Hz < f < .08 Hz) and several nuisance variables were removed by regression, including the following: (a) six movement parameters computed by rigid body translation and rotation during preprocessing, (b) mean whole-brain signal, (c) mean brain signal within the lateral ventricles, and (d) the mean signal within a deep WM ROI. Inclusion of the first temporal derivatives of these regressors within the linear model accounted for the time-shifted versions of spurious variance. For each voxel, a linear regression with the above model was applied to its fMRI signal time course. The residual of the regression was used to measure FC, which was defined as the correlation of residual fMRI signal time course between the mean of the ROI and each voxel. Correlation coefficients were converted to normally distributed Z-scores using the Fisher transformation and group-averaged results were reported as voxel-wise Z-scores. As a result, a FC connectivity map was built for each ROI, containing voxel-wise Z-score between the voxel and the average fMRI signal time course of the ROI. Finally, for each participant and each ROI, the FC disconnection score was computed as the sum of the z-scores in the ROI's FC connectivity map masked by the participant's lesion map.

Multivariate modeling and predictive analysis: Independent variables are lesion scores. Feature selection and training were based on the connectome-based predictive modeling algorithm. Performance was evaluated using AIC.
